# Trimethylamine N‐Oxide (TMAO), Inflammation, and Cardiovascular Risk Factors in Adults With Overweight and Obesity: Impact of Fresh Watermelon Consumption in a Randomized Controlled Crossover Trial

**DOI:** 10.1002/fsn3.72587

**Published:** 2026-11-14

**Authors:** Eryn M. Bollish, Emily Ager, Annie De Los Santos, Bermet Sydykova, Brian Nguyen, Michaela Renfro, Jesse Tanahara, Jacob Pascua, Mee Young Hong

**Affiliations:** ^1^ School of Exercise and Nutritional Sciences San Diego State University San Diego California USA

**Keywords:** cardiovascular risk factors, ICAM‐1, inflammation, obesity, TMAO, watermelon

## Abstract

Elevated trimethylamine N‐oxide (TMAO) levels are associated with increased cardiovascular disease (CVD) risk. Watermelon consumption has been shown to improve cardiovascular function by favorably modulating lipid profiles and endothelial function. However, its impact on circulating TMAO levels remains unknown. This study examined the effects of daily watermelon consumption on TMAO and other cardiovascular risk markers in adults with overweight and obesity. In this randomized crossover clinical trial, 32 participants (16 men, 16 women; mean age 25.6 ± 6.0 years; mean BMI 29.0 ± 3.7 kg/m^2^) consumed 330 g/day of fresh watermelon (100 kcal) or an isocaloric control (low‐fat cookies, LFC) for 4 weeks, separated by a 4‐week washout period. Body weight and BMI changed numerically during the watermelon intervention, but these changes were not statistically significant (*p* = 0.056 and *p* = 0.070, respectively). TMAO concentrations decreased significantly from baseline following watermelon consumption (*p* = 0.005); however, the difference between the watermelon and LFC interventions at week 4 was not statistically significant (*p* = 0.091). Triglycerides and LDL cholesterol were significantly reduced following the watermelon intervention (*p* < 0.05). ICAM‐1 levels were numerically, but not statistically significantly, lower after watermelon consumption (*p* = 0.083). Inflammatory markers TNF‐α and IL‐1β decreased significantly following the watermelon intervention (*p* < 0.05). Total antioxidant capacity (TAC) increased compared to LFC (*p* = 0.022). In conclusion, TMAO and selected cardiometabolic risk markers showed favorable within‐intervention changes during the watermelon period; however, the between‐intervention difference in TMAO was not statistically significant, although a numerical difference was observed. These exploratory findings require confirmation in larger, adequately powered studies.

**Trial Registration:** The study was registered at clinicaltrials.gov (NCT 06323070)

## Graphical abstract

In a randomized crossover trial, 32 adults with overweight or obesity consumed 330 g/day of watermelon (100 kcal) or isocaloric low‐fat cookies (LFC) for 4 weeks, separated by a 4‐week washout. Watermelon significantly reduced within‐intervention TMAO, triglycerides, LDL cholesterol, TNF‐α, and IL‐1β. Compared with LFC, the watermelon intervention resulted in lower LDH (lactate dehydrogenase) and higher total antioxidant capacity. Replacing a processed snack with fresh watermelon may favorably influence cardiovascular risk factors; however, larger, adequately powered studies are needed.

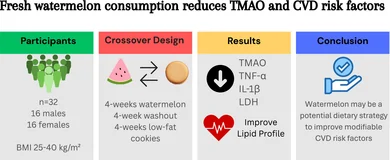

## 1 Introduction

Cardiovascular disease (CVD) is the leading cause of death worldwide, driven largely by modifiable factors such as poor diet and excess adiposity (WHO (World Health Organization) 2025). In the United States, 70%–75% of adults are overweight or obese, a condition strongly linked to hypertension, dyslipidemia, insulin resistance, and systemic inflammation—key mechanisms underlying CVD (Seravalle and Grassi 2017; Koliaki et al. 2019; Boutari et al. 2023). Extensive evidence indicates that diet is a critical determinant of cardiometabolic health and a target for CVD prevention (Chan et al. 2025).

Trimethylamine‐N‐oxide (TMAO), a gut microbiota–derived metabolite, has emerged as a biomarker of atherosclerosis, inflammation, and adverse cardiovascular outcomes (Verhaar et al. 2020; Li et al. 2022). Dietary nutrients are metabolized by gut microbes into trimethylamine (TMA), which is oxidized in the liver by flavin‐containing monooxygenase 3 (FMO3) to form TMAO. Circulating TMAO is primarily excreted by the kidneys. Diets rich in fiber and polyphenols can beneficially modulate gut microbiota, lower TMAO levels, and improve cardiometabolic outcomes (Sidhu et al. 2023; Lombardo et al. 2022; Crimarco et al. 2020). Fruit intake has been linked to reduced CVD risk in preclinical models, partly via TMAO‐related pathways (He et al. 2021; Kamkang et al. 2024).

Watermelon ( *Citrullus lanatus* ) is a low‐calorie, nutrient‐dense fruit containing lycopene and L‐citrulline, compounds shown to improve vascular function, lower blood pressure, and reduce oxidative stress (Luo et al. 2025; Hong et al. 2015; Zuraini et al. 2021). In animal studies, wild watermelon seed powder attenuated atherosclerosis and modulated circulating TMAO levels (Kaur et al. 2021, 2025). However, no human studies have examined the effect of fresh watermelon consumption on circulating TMAO. The objective of this study was to examine the effects of daily fresh watermelon consumption on TMAO concentrations (primary outcome) and other cardiovascular risk markers (secondary outcomes), including lipid profiles and inflammatory cytokines, in adults with overweight or obesity. We hypothesized that consuming 330 g/day of fresh watermelon would reduce circulating TMAO concentrations, improve lipid profiles, cell adhesion molecules, and liver function markers, and decrease inflammatory markers compared with an isocaloric low‐fat cookie (LFC) control. To our knowledge, this is the first human clinical trial to examine the effects of fresh watermelon consumption on TMAO and related cardiometabolic risk factors.

## 2 Materials and Methods

### 2.1 Participants

A total of 32 subjects (16 males and 16 females) participated in this study. Participants were recruited in 2024 through word of mouth and flyers posted throughout San Diego County. Inclusion criteria were healthy, sexually active, non‐smokers between the ages of 18–50 years with a BMI of 25–40 kg/m^2^. Participants with a wheat or watermelon allergy were excluded. Other exclusionary criteria for the study included the use of medications for blood pressure, cholesterol, diabetes, mental or sexual health. Participants taking nonprescription over‐the‐counter dietary supplements such as multivitamins were included and asked to maintain their supplementation regimen for the duration of the study. Female participants were excluded if they lacked a regular menstrual cycle or were pregnant or lactating. Continuous use of the same form of hormonal birth control in female participants was acceptable. Visits for female participants were scheduled during days 3–11 of their menstrual cycle to ensure consistency in hormones that could influence blood biomarkers. The study protocol was approved by the Institutional Review Board of San Diego State University (IRB number: HS‐2023‐0281). Each participant signed a consent form prior to the start of the study. This study is registered as clinical trial number (ClinicalTrials.gov Identifier: NCT06323070). Cardiometabolic markers were assessed as part of the broader evaluation of overall well‐being. These outcomes were not specifically designated as secondary outcomes in the original trial registration; the registration has subsequently been updated to include cardiometabolic outcomes as secondary outcomes. The completed CONSORT checklist for randomized crossover trials is provided in Supplementary Table 1.

### 2.2 Study Design

A crossover clinical trial was conducted. A randomized crossover design was used so that each participant received both interventions and served as their own control, thereby reducing interindividual variability and improving statistical efficiency. Participants were assigned to one of two intervention sequences using block randomization with a block size of five. The allocation sequence was generated by the principal investigator, who was blinded to participant identities. Because of the visible differences between watermelon and LFC, blinding of participants and study staff administering the interventions was not feasible. Participants were informed that the study compared two snack interventions without emphasizing watermelon as the intervention of interest. Laboratory technicians were blinded to treatment allocation.

During the intervention, participants consumed isocaloric (100 kcal) snacks of fresh watermelon (WM, 330 g) or six and a half low‐fat cookies (LFC, 22.7 g) (Amazon, Seattle, WA) daily for 4 weeks. The fresh watermelon contained 24.91 g carbohydrates (1.32 g of fiber and 20.46 g of sugar), 2.01 g protein and 0.49 g fat (0.05 g saturated, 0.12 g monounsaturated and 0.16 g polyunsaturated). The LFCs contained 16.71 g carbohydrate (0.43 g fiber and 8.51 g sugar), 1.13 g protein and 3.45 g fat (0.87 g saturated, 1.49 g monounsaturated, 0.88 g polyunsaturated). Half of the participants began with the WM intervention, while the other half began with the LFC intervention. To minimize potential carryover effects, participants completed a 4‐week washout period before crossing over to the alternate intervention. LFC snacks were prepackaged into single serving zip‐lock bags and provided to participants. Watermelon was pre‐cut and packaged in single‐serve deli containers. Weekly watermelon pickups were arranged during the watermelon intervention to ensure freshness. Participants were instructed to consume their assigned snack—watermelon (WM) or low‐fat cookies (LFC)—once daily while maintaining their usual diet and activity levels. Physical activity levels were obtained at each visit using a validated Physical Activity Recall Questionnaire (PAR‐Q) (Sallis et al. 1985). Participants were instructed to maintain their usual dietary habits throughout the study, consume only the watermelon provided during the WM intervention, and avoid watermelon during the LFC intervention.

Daily text reminders were sent to encourage compliance, and participants were asked to provide photographs of empty LFC bags or WM containers. Compliance was further confirmed using 24‐h dietary recalls. Four 24‐h dietary recalls were administered during each trial—twice at baseline and twice during the intervention—either in person or by telephone to assess adherence and potential compensatory dietary changes. Based on photographic confirmation and dietary recalls, 99% overall compliance with the intervention was achieved.

### 2.3 Anthropometrics

Height and weight were measured using a mechanical beam scale with a built‐in stadiometer (Detecto, Webb City, MO). Body fat was assessed during each visit utilizing a handheld bioelectrical impedance (BIA; Model HBFe306C, Omron, Lake Forest, IL) and dual‐energy x‐ray absorptiometry (DEXA, GE Healthcare Lunar Prodigy, Madison, WI). Waist and hip circumferences along with blood pressure (Model BP710N, Omron, Lake Forest, IL) were measured at each lab visit. Participants were instructed to fast for 12 h prior to each lab visit. Fasted blood draws were collected at the start of each lab visit and centrifuged at 1200 × *g* for 10 min at 4°C. Serum samples were stored at −80°C until analyzed.

### 2.4 Biochemical Analysis

#### 2.4.1 TMAO

There is a direct relationship between TMAO levels and the risk of major cardiovascular events. Trimethylamine N‐Oxide (TMAO) was measured using a competitive ELISA kit (MyBioSource Inc., San Diego, CA). A balance solution was applied to serum samples followed by the conjugate for antibody binding to amplify the measurement of TMAO concentration. Optical density was determined at 450 nm using a microplate reader.

All biochemical assays were performed in duplicate. The intra‐assay coefficient of variation (CV) for each biochemical measure was calculated by dividing the standard deviation (SD) of the replicate measurements by its mean and multiplying the result by 100.

#### 2.4.2 TAC

Total antioxidant capacity (TAC) was measured because of the demonstrated inverse relationship between antioxidant levels and major cardiovascular events. Cayman's Antioxidant Assay (Ann Arbor, MI) was used to measure the total antioxidant capacity of serum samples. The capacity of the antioxidants was compared with Trolox, a water‐soluble tocopherol analogue.

#### 2.4.3 TNF‐α

Tumor necrosis factor‐alpha (TNF‐α), a marker of inflammation, was quantified using the human TNF‐α ELISA kit. Upon addition of TMB, an initial blue color developed, which transformed into yellow upon the addition of acidic stop solution. Absorbance was read at 450 nm.

#### 2.4.4 C‐Reactive Protein (CRP)

CRP, an inflammatory marker, was measured because of the connection between inflammation and cardiovascular diseases. CRP was measured using an ELISA kit (Immundiagnostik AG, Bensheim, Germany). A peroxidase‐labeled detection antibody was used for CRP detection to catalyze the conversion of the substrate TMB (3,3′, 5,5′‐tetramethylbenzidine). The addition of an acidic stopping solution produces a yellow color. The intensity of the yellow color is directly proportional to the concentration of CRP in the sample. Absorbance was read at 450 nm with a spectrophotometer.

#### 2.4.5 Lipid Profiles

Blood lipid profiles, including triglycerides (TG), total cholesterol (TC), and high‐density lipoprotein cholesterol (HDL‐C) were analyzed using colorimetric assay kits (Stanbio Laboratory, Boerne, TX). Low‐density lipoprotein cholesterol (LDL‐C) was calculated using the following formula: LDL‐C = TC – (HDL‐C) – (TG/5) (Friedewald et al. 1972).

#### 2.4.6 IL‐1β

IL‐1β is a pro‐inflammatory cytokine contributing to inflammation and the subsequent development of cardiovascular disease. IL‐1β was measured using a Quantikine immunoassay (R&D Systems, Minneapolis, MN). An enzyme‐linked polyclonal antibody with the addition of substrate initially resulted in the development of a blue color, which turned yellow upon the addition of the stop solution. Absorbance was read at 450 nm.

#### 2.4.7 Liver Function Markers

Aspartate aminotransferase (AST), alanine aminotransferase (ALT), lactate dehydrogenase (LDH), alkaline phosphatase (AP), creatine kinase (CK), and gamma‐glutamyl transferase (γ‐GT) activities were measured using EKF Diagnostics assay kits (Boerne, TX). Absorbance for AST, ALT, LDH, and CK was read at 340 nm whereas AP and γ‐GT were read at 405 nm.

#### 2.4.8 VCAM‐1, ICAM‐1, E‐selectin and P‐selectin

VCAM‐1, ICAM‐1, E‐selectin, and P‐selectin levels were measured using ELISA kits (VCAM‐1: Invitrogen, Waltham, MA; ICAM‐1, E‐selectin, and P‐selectin: R&D Systems, Minneapolis, MN) according to the manufacturers' instructions. All measurements utilized HRP‐conjugated antibodies, and absorbance was read at 450 nm using a spectrophotometer.

### 2.5 Statistical Analysis

Because published data on the effects of watermelon consumption on TMAO were unavailable when the study was designed, the a priori sample size calculation was based on LDL‐cholesterol outcomes previously reported in adults with overweight or obesity (Lum et al. 2019). Assuming an effect size of 0.53, a two‐sided *α* of 0.05, and 80% power, a minimum of 30 participants was required for this crossover trial. The final sample included 32 participants.

Data were analyzed using SPSS version 26 (IBM, Armonk, NY). Repeated measures ANOVA and paired *t*‐tests were conducted to analyze intervention effects. Baseline differences between trials were assessed using paired *t*‐tests. When baseline differences were significant, follow‐up analyses were conducted using analysis of covariance (ANCOVA) with baseline values as covariates. Mixed ANOVAs were conducted to examine interactions with sex. The results were primarily reported for the combined sample because stratification by sex would further reduce statistical power. Sex‐specific results were presented only when different patterns were observed between male and female participants and should also be interpreted as exploratory. Separate linear mixed‐effects models were used to examine the associations of TMAO with total antioxidant capacity (TAC) and each inflammatory marker (TNF‐α, IL‐1β, and CRP), while accounting for repeated measurements within participants across trials and time points. Potential carryover effects were assessed using a linear mixed‐effects model that included treatment, period, sequence, and treatment‐by‐period interaction as fixed effects. All data are presented as means ± standard deviations (SDs), with a *p* ≤ 0.05 considered statistically significant. No adjustment for multiple comparisons was applied; therefore, all secondary outcome analyses should be considered exploratory. Significant treatment effects are presented as mean differences with corresponding 95% confidence intervals, in addition to *p* values.

## 3 Results

### 3.1 Participants

A total of 76 participants were assessed for eligibility. Among the 43 participants excluded, 14 had a BMI below 25 kg/m^2^ and 29 were unable to complete the study due to time constraints or other personal reasons. Thirty‐three participants were allocated to the intervention. One male participant withdrew after the first visit due to personal reasons. Therefore, a total of 32 participants (16 males, 16 females) completed the study (Figure 1).

**FIGURE 1:**
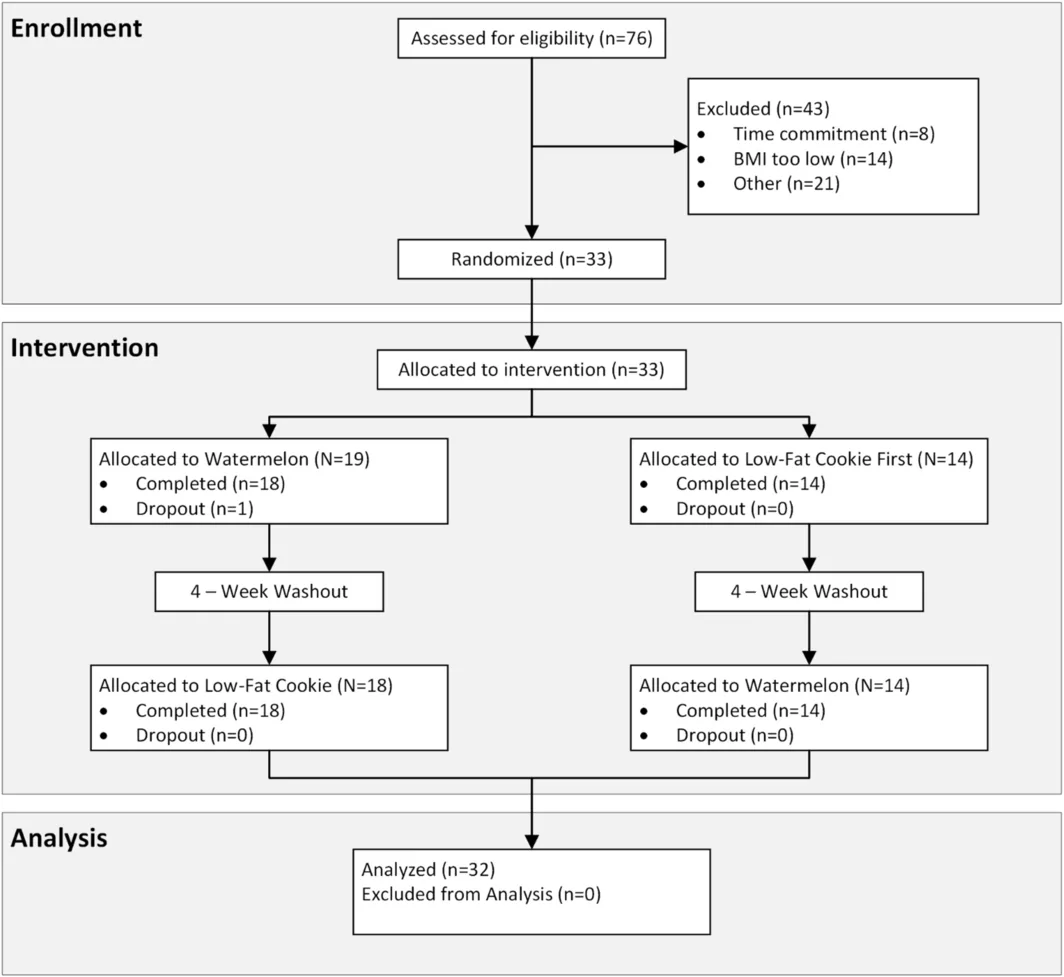
CONSORT flow diagram of participant selection.

Participants ranged in age from 19 to 43 years, with a mean age of 25.63 ± 5.98 years. The average age of male participants was 24.50 ± 4.83 years, while the average age of female participants was 26.75 ± 6.92 years. Ethnic composition included 10 White, 5 Black, 14 Hispanic, and three Asian participants. No adverse events, unintended effects, or intervention‐related withdrawals were reported during either the watermelon or LFC intervention period.

### 3.2 Anthropometrics

Body weight showed a non‐significant trial‐by‐time interaction (*p* = 0.056), with a numerical but non‐significant increase following LFC consumption and a decrease following WM consumption (Table 1). Among males, a significant trial‐by‐time interaction was observed (*p* = 0.050); body weight increased significantly following LFC consumption (*p* = 0.037) but did not change significantly following WM consumption. Among females, no significant within‐ or between‐intervention differences in body weight were observed. Similarly, a non‐significant trial‐by‐time interaction was observed for overall BMI (*p* = 0.070). BMI did not change significantly among females, whereas males showed a numerical but non‐significant increase following LFC consumption (*p* = 0.052).

**TABLE 1:** Participant anthropometric measurements at baseline and week 4 of intervention.

Measurements	Watermelon (WM)		Low‐fat cookies (LFC)	
	Baseline	Week 4	Baseline	Week 4
Body weight (kg)	84.98 ± 14.48	84.63 ± 14.70*	84.71 ± 14.21	84.97 ± 14.45*
BMI	29.07 ± 3.71	28.95 ± 3.84*	29.00 ± 3.74	29.08 ± 3.82*
SBP (mmHg)	118.66 ± 13.30	121.75 ± 12.38	118.88 ± 12.76	118.84 ± 14.25
DBP (mmHg)	75.59 ± 8.03	75.78 ± 8.83	76.25 ± 9.76	76.53 ± 8.00
W/H ratio	0.815 ± 0.07	0.811 ± 0.06*	0.816 ± 0.06	0.819 ± 0.06
Body fat‐BIA (%)	30.26 ± 10.07	29.98 ± 9.95	30.07 ± 9.99	29.96 ± 9.70
Body fat‐DXA (%)	34.64 ± 9.89	34.98 ± 9.84	34.77 ± 9.86	34.75 ± 9.88

*Note:* Data are presented as means ± SDs. *N* = 32.

Abbreviations: BIA, bioelectrical impedance analysis; BMI, body mass index; DBP, diastolic blood pressure; DXA, dual x‐ray absorptiometry; SBP, systolic blood pressure; W/H, waist‐to‐hip ratio.

* Nonsignificant numerical differences with 0.05 < *p* < 0.10.

Waist circumference showed a non‐significant trial‐by‐time interaction (*p* = 0.062), with a numerical but non‐significant increase following LFC consumption (*p* = 0.098) and a decrease following WM consumption (*p* = 0.081). At week 4, waist circumference was significantly lower following WM consumption than following LFC consumption (*p* = 0.023). Hip circumference did not differ significantly within or between interventions. The waist‐to‐hip ratio was numerically lower after 4 weeks of WM consumption than after LFC consumption, although the difference was not statistically significant (*p* = 0.071). No significant differences were observed in SBP, DBP, or body fat percentage.

### 3.3 Lipid Profile

Triglycerides and LDL‐C levels significantly decreased after 4 weeks of WM consumption compared to baseline (*p* = 0.045 and *p* = 0.035, respectively; Figure 2). Triglyceride levels were numerically, but not statistically significantly, lower following WM consumption than following LFC consumption (*p* = 0.071). Following the WM intervention, TC decreased numerically (*p* = 0.071), whereas HDL‐C increased numerically from baseline (*p* = 0.061); however, neither change was statistically significant.

**FIGURE 2:**
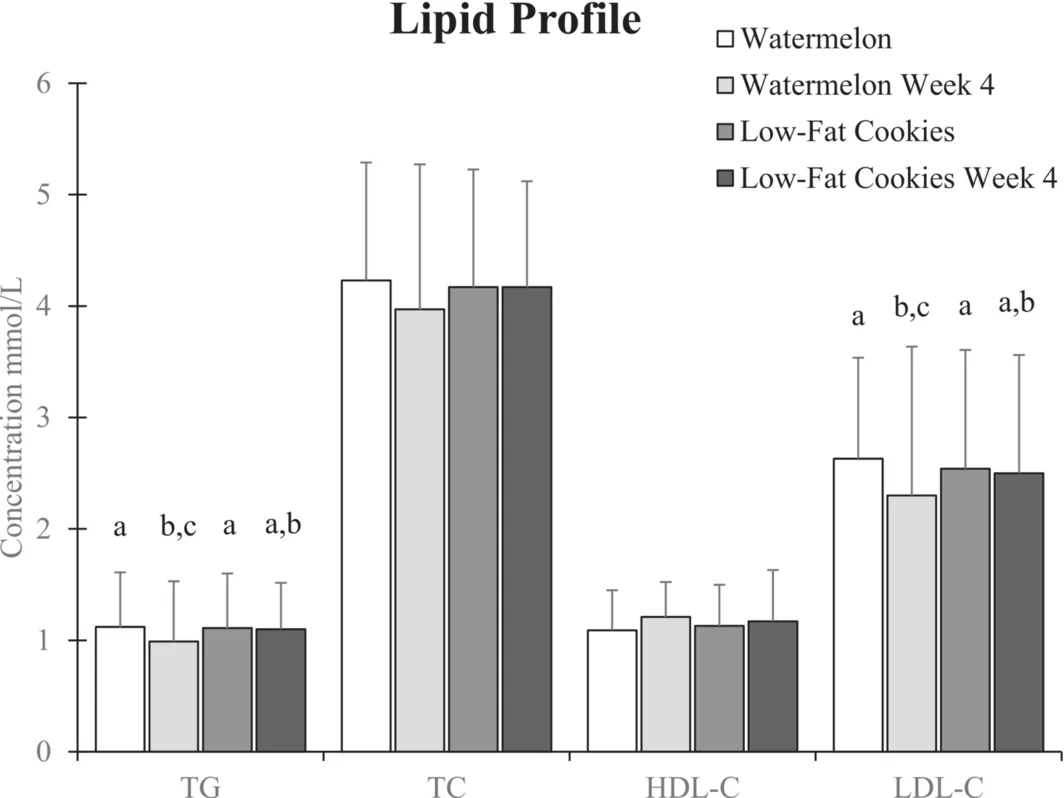
Effects of watermelon (WM) and low‐fat cookie (LFC) consumption on serum lipid values at baseline and week 4 of each intervention. Data are presented as means ± SDs. *N* = 32. Within a variable, values with different letters are statistically different at *p* ≤ 0.05. Triglycerides (TG), total cholesterol (TC), high‐density lipoprotein cholesterol (HDL‐C), and low‐density lipoprotein cholesterol (LDL‐C). Intra‐assay CV of TG was 9.96, TC 9.72, and HDL‐C 7.54.

### 3.4 TMAO

A significant trial‐by‐time interaction was observed for serum TMAO levels (*p* = 0.003; Table 2). TMAO levels significantly decreased from baseline after 4 weeks of WM consumption (mean difference = 6.604 μg/L; 95% CI, 2.126–11.082; *p* = 0.005), while a non‐significant numerical increase in TMAO level from baseline was noted after LFC consumption (*p* = 0.064). However, the post‐intervention difference between the watermelon and LFC interventions did not reach statistical significance (*p* = 0.091). No statistically significant sequence effects were observed for TMAO, which may suggest that the analysis did not detect evidence of a carryover effect.

**TABLE 2:** Serum inflammation markers at baseline and week 4 of intervention.

Serum marker	Watermelon (WM)		Low‐fat cookies (LFC)	
	Baseline	Week 4	Baseline	Week 4
TMAO (μg/L)	147.3 ± 25.83^b^	140.7 ± 18.58^a^	140.2 ± 24.28^a^	144.7 ± 20.25^a,b^
CRP (mg/L)	2.86 ± 2.42	2.99 ± 2.53	2.39 ± 1.95	2.91 ± 2.44*
TNF‐α (pg/mL)	31.29 ± 27.06^a^	26.04 ± 17.51^b^	33.79 ± 33.15^a^	29.38 ± 26.53^a,b^
IL‐1β (pg/mL)	2.36 ± 1.36^a^	1.77 ± 0.40^b^	2.16 ± 0.95^a,b^	2.15 ± 1.30^a,b^

*Note:* Data are presented as means ± SDs. *N* = 32. Within a variable, values with different superscript letters are statistically significant at *p* ≤ 0.05. Data within rows with varying superscript letters are significantly different at *p* ≤ 0.05. Intra‐assay CV of TMAO was 9.81, CRP 4.20, TNFα 10.38, and IL‐1β 2.80.

Abbreviations: CRP, C‐reactive protein; IL‐1β, interleukin‐1 beta; TMAO, trimethylamine N‐oxide; TNF‐α, tumor necrosis factor alpha.

* Nonsignificant numerical differences with 0.05 < *p* < 0.10.

### 3.5 Inflammation Markers

CRP levels increased numerically following LFC consumption, although the change was not statistically significant (*p* = 0.055), whereas no significant change was observed following WM consumption (Table 2). TNF‐α levels decreased significantly from baseline after 4 weeks of WM consumption (mean difference = 5.434 pg/mL; 95% CI, 0.187–10.688; *p* = 0.029). Similarly, IL‐1β levels decreased significantly from baseline following WM consumption (mean difference = 0.586 pg/mL; 95% CI, 0.083–1.089; *p* = 0.024) and were numerically, but not statistically significantly, lower at week 4 following WM consumption than following LFC consumption (*p* = 0.056).

### 3.6 Liver Function Markers

There was a significant increase in LDH levels between baseline and week 4 of LFC consumption (mean difference = −35.757 U/L; 95% CI, −66.749 to −4.764; *p* = 0.025) (Table 3). There was a significant difference between week 4 LFC consumption and week 4 watermelon consumption, with watermelon having a lower mean LDH level (mean difference = 31.543 U/L; 95% CI, 0.800–62.286; *p* = 0.028). Levels of AST, ALT, AP, and CK were all found to have statistically insignificant differences. Because 𝛾‐GT baseline values differed between groups, data were analyzed using ANCOVA; 𝛾‐GT values remained insignificant after adjusting for baseline as a covariate.

**TABLE 3:** Liver function enzymes and cellular adhesion molecules at baseline and week 4 of intervention.

	Watermelon (WM)		Low‐fat cookies (LFC)	
	Baseline	Week 4	Baseline	Week 4
Liver marker				
AST (U/L)	11.13 ± 6.86	12.04 ± 5.50	13.37 ± 7.58	14.21 ± 15.48
ALT (U/L)	10.30 ± 5.12	11.48 ± 6.01	11.28 ± 5.54	13.19 ± 9.12
LDH (U/L)	174.55 ± 45.52^a^	180.32 ± 56.18^a^	175.31 ± 57.70^a^	211.06 ± 68.79^b^
AP (U/L)	78.39 ± 19.42	76.49 ± 20.12	77.13 ± 22.85	73.55 ± 22.02
CK (U/L)	93.47 ± 101.16	89.15 ± 77.56	119.43 ± 151.55	108.64 ± 138.90
γ‐GT (U/L)	16.81 ± 8.64	17.99 ± 10.59	19.10 ± 11.19	18.56 ± 9.69
CAM marker				
VCAM‐1 (ng/mL)	968.4 ± 638.0	1101.1 ± 1000.7	959.1 ± 484.7	1110.9 ± 693.8
ICAM‐1 (ng/mL)	127.2 ± 55.4	122.1 ± 41.6*	133.8 ± 55.7	134.0 ± 62.3
E‐selectin (ng/mL)	2.7 ± 2.6	2.6 ± 2.3	2.8 ± 2.4	2.6 ± 2.5
P‐selectin (ng/mL)	89.6 ± 42.2	93.4 ± 41.1	97.1 ± 40.8	95.8 ± 43.1

*Note:* Data are expressed as means ± SDs. *N* = 32. Within a variable, values with different superscript letters are statistically significant at *p* ≤ 0.05. Intra‐assay CV of AST was 9.08, ALT 9.88, LDH 10.27, AP 10.49, CK 10.44, r‐GT 10.06, VCAM‐1 9.72, ICAM‐1 3.36, E‐selectin 4.25, and P‐selectin 9.47.

Abbreviations: γ‐GT, gamma‐glutamyl transferase; ALT, alanine aminotransferase; AP, alkaline phosphatase; AST, aspartate aminotransferase; CK, creatine kinase; ICAM‐1, intercellular adhesion molecule 1; LDH, lactate dehydrogenase; VCAM‐1, vascular cell adhesion molecule 1.

* Nonsignificant numerical differences with 0.05 < *p* < 0.10.

### 3.7 VCAM‐1, ICAM‐1, E‐selectin and P‐selectin

No significant within‐ or between‐intervention differences were observed in VCAM‐1 levels (Table 3). After 4 weeks, serum ICAM‐1 levels were numerically lower following WM consumption than following LFC consumption, although the difference was not statistically significant (*p* = 0.083). No significant within‐ or between‐intervention differences were observed in E‐selectin or P‐selectin levels.

### 3.8 Total Antioxidant Capacity

Total antioxidant capacity (TAC) levels increased numerically from baseline following WM consumption (*p* = 0.088; Figure 3), with no significant change following LFC consumption. At week 4, TAC levels were significantly higher following WM consumption than following LFC consumption (mean difference = −0.159 mM; 95% CI, −0.294 to −0.024; *p* = 0.022).

**FIGURE 3:**
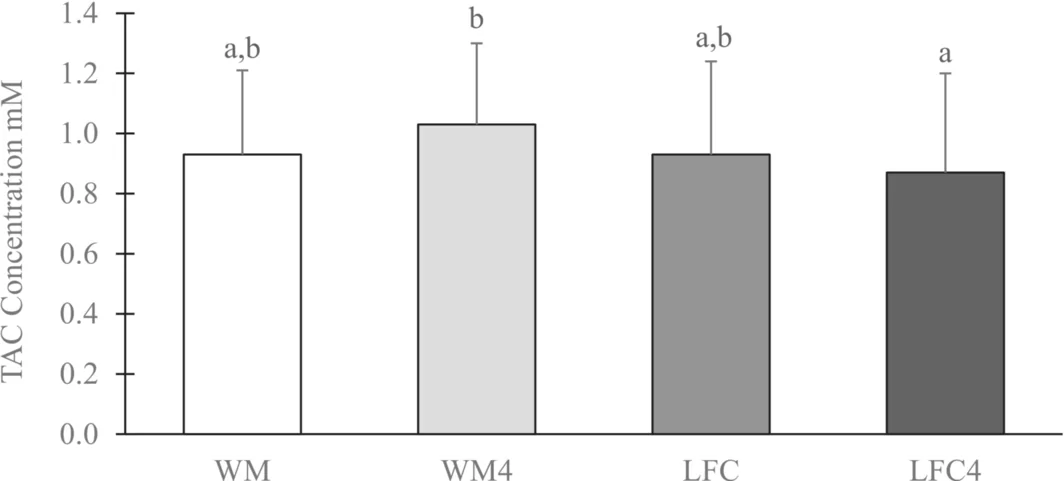
Effects of watermelon (WM) and low‐fat cookie (LFC) consumption on serum values of total antioxidant capacity (TAC) at baseline and week 4 of each intervention. Data are presented as means ± SDs. *N* = 32. Values with different letters are statistically different at *p* ≤ 0.05. Intra‐assay CV of TAC was 8.92.

### 3.9 Correlations Between TMAO, Inflammation Markers and TAC

TMAO was positively associated with TNF‐α (*B* = 0.415, *p* = 0.001) and CRP (*B* = 1.366, *p* = 0.043). Among male participants, TMAO was significantly and positively associated with IL‐1β (*B* = 3.029, *p* = 0.040). In the combined male and female data, TNF‐α was significantly and positively associated with IL‐1β (*B* = 6.729, *p* < 0.001).

## 4 Discussion

Watermelon ( *Citrullus lanatus* ) is a widely consumed fruit rich in water, antioxidants, and bioactive compounds. This study examined the effects of daily fresh watermelon consumption versus an isocaloric amount of LFC on cardiovascular risk markers, including circulating TMAO, lipid profiles, cell adhesion molecules, and inflammatory markers. To our knowledge, this is the first human study to examine the impact of fresh watermelon on TMAO levels.

Our results demonstrated a within‐intervention reduction in blood TMAO following 4 weeks of watermelon intake compared to baseline in adults with overweight or obesity. While no human studies have directly assessed watermelon's effects on TMAO, watermelon seed powder has been shown to modulate TMAO in LDL receptor–knockout mice (Kaur et al. 2021, 2025).

Previous studies have proposed that antioxidant activity may influence TMAO metabolism through gut microbiota or hepatic FMO3 activity (Chen et al. 2016; Coutinho‐Wolino et al. 2021). Watermelon is rich in antioxidants (Kim et al. 2024; Oms‐Oliu et al. 2009), and its consumption has been associated with increased serum total antioxidant capacity (TAC) in humans (Shanely et al. 2016). Consistent with previous reports (Kim et al. 2024; Oms‐Oliu et al. 2009; Shanely et al. 2016), TAC was higher following watermelon consumption than following the control intervention in our study. However, the inverse correlation between TAC and TMAO was not statistically significant, and gut microbiota composition, TMA production, and FMO3 activity were not assessed. Therefore, the mechanisms underlying the observed findings require further investigation.

Lycopene, a carotenoid abundant in watermelon, has been hypothesized to attenuate TMAO concentrations and TMAO‐related damage through activation of the AMPK/SIRT1 pathway and reductions in oxidative stress and cellular senescence (Liu et al. 2025). Watermelon juice consumption has also been shown to increase circulating lycopene concentrations in older women (Kim et al. 2024). However, these pathways were not assessed in the present study.

Watermelon is also a rich natural source of L‐citrulline, which can be converted to L‐arginine and subsequently to nitric oxide (NO), potentially supporting vascular function (Luo et al. 2025; Hong et al. 2015; Zuraini et al. 2021). Although no association between citrulline and TMAO was observed in one study of patients with CVD (Konieczny et al. 2022), L‐arginine supplementation reduced TMAO concentrations in a randomized controlled trial involving adults with overweight and cardiometabolic risk factors (Dimina et al. 2023). In hypertensive rats, TMAO was reported to inhibit L‐arginine at its catalytic site, thereby reducing aortic NO production (Martelli et al. 2025). Collectively, these findings raise the possibility that watermelon constituents, including lycopene and L‐citrulline, may be relevant to biological pathways associated with TMAO‐related endothelial dysfunction. However, these mechanisms were not directly investigated in the present study. Further mechanistic studies are needed to directly evaluate these potential pathways.

Inflammation is central to the initiation and progression of CVD, contributing to endothelial dysfunction and thrombotic events. TMAO has been shown to promote TNF‐α‐induced thrombotic factor expression in vascular endothelial cells in patients with acute myocardial infarction (Cheng et al. 2019). Elevated levels of TMAO in obese mice were associated with increased expression of pro‐inflammatory cytokines TNF‐α and IL‐1β in kidney tissue (Sun et al. 2017). In our study, watermelon consumption significantly reduced TNF‐α and IL‐1β with positive correlations between TMAO and these cytokines, suggesting that reduced inflammation may be associated with lower TMAO levels.

Several animal and human studies have reported favorable changes in lipid profiles following watermelon consumption (Hong et al. 2015; Lum et al. 2019; Lucas et al. 2020; Massa et al. 2016). A recent meta‐analysis of randomized controlled trials found reductions in total cholesterol (TC) and LDL cholesterol following watermelon consumption in humans (Karimi et al. 2023). Potential mechanisms have been proposed based primarily on animal studies. In rats, watermelon supplementation altered the expression of hepatic genes involved in lipid synthesis, including FAS, HMGCR, SREBP1, and SREBP2, accompanied by reductions in triglycerides, LDL cholesterol, and TC (Hong et al. 2018). In high‐fat‐diet‐fed Wistar rats, watermelon extract reduced PCSK9 expression and increased ABCA1 expression, accompanied by decreased TC, triglycerides, and LDL cholesterol and increased HDL cholesterol (Bahrudin et al. 2025). In the present study, reductions in LDL cholesterol and triglycerides were observed during the watermelon intervention, along with nonsignificant numerical decreases in TC and increases in HDL cholesterol, although these changes were not statistically significant. Because these molecular pathways were not assessed in our study, their potential contribution to the observed changes remains hypothetical and requires further investigation.

Beyond biochemical markers, watermelon's high water content promotes satiety and may support weight management. Lum et al. (Lum et al. 2019) demonstrated that daily fresh watermelon intake for 4 weeks reduced body weight and BMI in adults with overweight or obesity. Similarly, an eight‐week study in children with overweight and obesity showed that blended watermelon intake significantly decreased body weight and body fat percentage (Daughtry et al. 2023). L‐citrulline, abundant in watermelon, has been shown to decrease body weight and fat mass in obese mice (Kudo et al. 2017) and inhibit weight gain and hepatic fat accumulation by enhancing lipid metabolism and fatty acid β‐oxidation in SHRSP5/Dmcr rats (Kudo et al. 2021).

Lactate dehydrogenase (LDH) concentrations are elevated in patients with coronary heart disease and have been positively associated with coronary artery stenosis (Tan et al. 2021). In the present study, serum LDH was significantly lower following watermelon consumption than following the LFC intervention, whereas other liver‐associated enzymes remained unchanged. Although this finding may indicate a potential benefit, LDH is a nonspecific marker and the underlying mechanism remains unclear. Arginine has been proposed to enhance NO production, support NO‐dependent vasodilation, and reduce cellular damage (Nikolic et al. 2007; Loscalzo 2000), while watermelon seed extract has been shown to lower LDH concentrations in diabetic rats (Karikpo et al. 2018). However, these mechanisms were not evaluated in the present study and should be considered hypothetical.

Intercellular adhesion molecule‐1 (ICAM‐1) is a marker of endothelial inflammation and has been positively associated with coronary artery stenosis in patients with coronary heart disease (Tan et al. 2021). In the present study, ICAM‐1 concentrations were numerically lower following watermelon consumption than following the LFC intervention, although the difference was not statistically significant. Arginine availability has been inversely associated with the expression of adhesion molecules, including ICAM‐1, potentially through modulation of pro‐inflammatory transcription factors such as nuclear factor‐κB (NF‐κB) (Yeh et al. 2007; Adams, Jessup, et al. 1997; Adams, McCredie, et al. 1997). Because watermelon is rich in citrulline, a precursor to arginine, this pathway represents a possible mechanism through which watermelon could influence endothelial inflammatory markers, although further investigation is needed to confirm this mechanism.

Several limitations warrant consideration. First, the relatively small sample size may have limited the statistical power to detect modest between‐intervention differences, particularly for secondary outcomes. In addition, the large number of outcomes and statistical comparisons increased the possibility of chance findings. The 4‐week intervention period may also have been insufficient to detect longer‐term changes in TMAO and other cardiometabolic outcomes. Therefore, the findings should be considered exploratory and interpreted with caution. Larger, longer‐term, adequately powered studies with prespecified primary outcomes and appropriate consideration of multiple testing are needed.

TMAO concentrations were measured using a competitive ELISA rather than a mass spectrometry‐based method. Although ELISA provides a practical approach for TMAO assessment, it may have lower analytical specificity and sensitivity than LC–MS/MS. Therefore, future studies using mass spectrometry‐based methods are warranted to confirm these findings. Furthermore, because TMAO is influenced by gut microbiota composition, the potential microbiome‐mediated effects of watermelon remain unclear (Witkowski et al. 2020). Although whole‐watermelon consumption has been shown to alter gut microbiota composition in mice with obesity (Becraft et al. 2020), these potential effects require further investigation in humans.

Although the LFC intervention provided a practical, isocaloric, real‐world snack comparison, it was not an inert or nutritionally matched control and differed from watermelon in water, fiber, sugar composition, micronutrients, and bioactive compounds. Therefore, the study evaluates the effect of replacing a processed snack with watermelon rather than the isolated effect of watermelon or its specific bioactive compounds. Future studies using a more closely matched comparator are needed to better isolate the effects of watermelon consumption.

Habitual dietary intake, including usual fruit and vegetable consumption, was not comprehensively assessed at baseline. Although participants were instructed to maintain their usual dietary habits and avoid watermelon outside the assigned intervention, unmeasured dietary factors or changes in food intake may have influenced the outcomes. Thus, potential confounding by other dietary components cannot be excluded. In addition, adherence was assessed primarily through indirect measures. Although reported compliance was high, these methods cannot fully exclude unreported deviations from the intervention.

This study included a 4‐week washout period. Baseline TMAO concentrations did not differ significantly between the two intervention periods, and no statistically significant sequence effect was observed for TMAO. However, given the exploratory nature of the analysis, the possibility that residual intervention effects or intervention order influenced the findings cannot be completely excluded.

Finally, participants were overweight or obese but were generally metabolically healthy and had relatively low baseline TMAO concentrations. These characteristics limit the generalizability of the findings to other populations, including older adults and individuals with established CVD, chronic kidney disease, elevated TMAO concentrations, or greater cardiometabolic risk. Further studies in these populations are warranted.

## 5 Conclusion

Our exploratory findings suggest that daily consumption of fresh watermelon was associated with favorable within‐intervention changes in selected CVD risk factors, including circulating TMAO, lipid profiles, and inflammatory cytokines. Although a numerical difference in TMAO was observed between the interventions, it did not reach statistical significance. Replacing a commonly consumed processed snack with fresh watermelon may have the potential to favorably influence obesity‐associated CVD risk factors; however, these preliminary findings require confirmation in larger, adequately powered studies.

## Author Contributions

**Eryn M. Bollish:** investigation, writing – original draft. **Emily Ager:** investigation, project administration. **Annie De Los Santos:** investigation. **Bermet Sydykova:** investigation. **Brian Nguyen:** investigation. **Michaela Renfro:** investigation. **Jesse Tanahara:** investigation. **Jacob Pascua:** investigation. **Mee Young Hong:** conceptualization, investigation, funding acquisition, data curation, formal analysis, supervision, resources, project administration, writing – original draft, writing – review and editing.

## Funding

This work was supported by the National Watermelon Promotion Board [DTD 11–03‐23].

## Conflicts of Interest

The authors declare no conflicts of interest.

## Supporting information


**Supplementary Table 1:** CONSORT checklist of information to include when reporting randomized crossover trials.

## Data Availability

The data that support the findings of this study are available from the corresponding author upon reasonable request.
